# Primary closure versus the “Volcano” technique (PRIVEVO): a prospective observational study of purse-string wound closure for stoma reversal

**DOI:** 10.1186/s12893-026-03519-6

**Published:** 2026-01-24

**Authors:** Péter Kolozsi, Kitti Nagy, Tamás Felföldi, Zsolt Varga, Sándor Kovács, Dávid Ágoston Kovács, Dezső Tóth

**Affiliations:** 1 Institute for Surgery, University of Debrecen, Móricz Zsigmond krt 22, Debrecen, 4032 Hungary; 2 Faculty of Economics and Business, Coordination and Research Centre for Social Sciences, University of Debrecen, Debrecen, Hungary

**Keywords:** Colostomy, Ileostomy, Stoma closure, Purse-string, Colorectal cancer, Hartmann’s procedure, Stoma takedown, Stoma reversal

## Abstract

**Background:**

Stoma creation is an essential part of general surgery, used in procedures such as protective loop ileostomy formed during low rectal resection or coloanal anastomosis, and in Hartmann’s procedures, often performed in emergency settings. Despite its clinical importance, the optimal wound closure technique following stoma reversal remains controversial. This study aims to identify a more effective wound closure method following stoma takedown.

**Methods:**

Between June 1, 2017, and January 31, 2020, we prospectively observed 75 patients at the University of Debrecen, Hungary, who underwent stoma closure (29 colostomies and 46 ileostomies). Among these patients, 37 underwent purse-string “Volcano” suture closure (VSC), while 38 underwent primary suture closure (PSC). We recorded demographic data, operative times, and postoperative outcomes, including wound infections. The primary endpoint was the rate of surgical site infection (SSI) in the short-term and the occurrence of postoperative hernia in the long-term period. Secondary endpoints included operative time, duration of wound dressing, and surgical complications according to the Clavien–Dindo classification. The planned follow-up period for long-term complications was 5 years. In this study, Surgical Site Infection (SSI) was defined and classified strictly according to the Centers for Disease Control and Prevention (CDC) Guideline for the Prevention of Surgical Site Infection (2017).

**Results:**

No significant differences were observed between the VSC and PSC groups in age, sex, BMI, comorbidities, ASA classification, time from primary surgery to stoma closure, or length of hospital stay. However, the operative time was significantly shorter in the VSC group (59 min) compared to the PSC group (87 min; *p* = 0.034). Notably, the incidence of postoperative wound infection was 0% in the VSC group and 18.4% in the PSC group respectively (*p* = 0.012).

**Conclusion:**

Our prospective observational study demonstrates that the “Volcano” (purse-string) wound closure technique provides significant advantages over conventional primary closure, notably lowering surgical site infection rates and operative time. Based on these results, we recommend the Volcano technique for stoma reversal following both ileostomy and colostomy.

**Supplementary Information:**

The online version contains supplementary material available at 10.1186/s12893-026-03519-6.

## Introduction

Stoma formation remains a fundamental component of modern surgical practice. According to 2022 data, approximately one million individuals in the United States live with a stoma, with an additional 100,000 ostomies being performed annually [1]. Ostomy procedures can be categorized by type (ileostomy vs. colostomy), urgency (emergency vs. elective), and indication (benign vs. malignant disease). Among emergency stomas, Hartmann’s procedure–a single-barrel end colostomy–is commonly performed to treat complicated left-sided colon diseases [2], though it is also utilized in select elective cases. Elective diverting loop ileostomy is also increasingly performed due to the growing prevalence of sphincter-preserving colorectal procedures such as low anterior resection and coloanal anastomosis [3].

Despite their clear benefits in mitigating the clinical consequences of complications such as anastomotic leakage, stomas can present challenges for both surgeons and patients [3, 4]. Difficulties can arise not only while living with a stoma but also during its closure. Common complications after stoma reversal include hand-sewn or stapled anastomotic leaks, as well as incisional hernias occurring in approximately 2–12% of cases [5, 6]. However, the most frequent complication is surgical site infection (SSI), with reported rates ranging from 2% to 41% [6–8]. To address this, Banerjee introduced a one-layer purse-string technique in 1997 [9], showing potential for reduced SSI rates and improved cosmetic outcomes [5, 10].

Recognizing that both fascial and skin closure are critical to reducing postoperative morbidity, Krenzien and colleagues described a modified Banerjee closure, the so-called “Vulkan” or “Volcano” technique in 2017 [11]. Their approach employs consecutive circular subcutaneous sutures in three layers, with the first layer incorporating the fascial layer of the rectus abdominis to minimize the dead space in which fluid can accumulate. Similar to Banerjee’s and Krenzien’s methods, the “gunsight” closure described by Lim partially closes the stoma site while allowing external drainage. This approach has also shown promising results in minimizing SSI and may offer potentially improved cosmetic outcomes [12, 13]. The primary aim of this study is to compare the efficacy of the “Volcano” technique versus primary suture closure regarding surgical site infection rates in the short term and the occurrence of postoperative hernia formation in the long term.

## Materials and methods

### Ethical statement and patient consent

The study protocol was reviewed and approved by the Regional and Institutional Research Ethics Committee of the Clinical Center of the University of Debrecen (decision DE RKEB/IKEB 6827 − 2024). Informed consent was obtained from all individual participants included in the study. All procedures were performed in accordance with the ethical standards of the institutional research committee and with the 1964 Helsinki Declaration and its later amendments.”

### Patients

This article presents the primary results of the Primary Closure Versus the Volcano Technique Stoma Closure (PriVeVo) prospective observational study. We enrolled all patients over 18 years of age who underwent stoma closure between June 1, 2017, and January 31, 2020, at the Surgery Clinic of the University of Debrecen, Hungary. Both colostomy and ileostomy closures were included, regardless of underlying etiology (benign or malignant). A total of 75 patients (29 colostomy reversals and 46 ileostomy reversals) were included. Among these, 37 patients received the Volcano (purse-string) suture closure (VSC; 12 colostomies and 25 ileostomies) and 38 patients underwent primary suture closure (PSC; 17 colostomies and 21 ileostomies).

### Surgical techniques

All patients received a single prophylactic antibiotic dose 30 min before the procedure (2 g of a third-generation cephalosporin plus 500 mg metronidazole intravenously). No additional antibiotics were administered postoperatively. In colostomy cases, we performed a median laparotomy to fashion a colorectal anastomosis using a circular stapler. For ileostomy reversals, after resecting the stoma, a hand-sewn side-to-side ileo-ileostomy was created. Usually, no midline laparotomy was needed for ileostomy closures.

After performing the anastomosis, the abdominal wall was reconstructed using interrupted polyglactin sutures and irrigated with diluted povidone-iodine. In the case of primary closure (PSC), the subcutaneous tissues were approximated with interrupted 2 − 0 polyglactin sutures, and the skin was closed with interrupted 3 − 0 polypropylene sutures. In the case of Volcano (purse-string) closure (VSC), a three-layered purse-string closure was performed with 2 − 0 polyglactin sutures: (1) deep subcutaneous (including the fascial layer of rectus abdominis), (2) superficial subcutaneous, and (3) subcuticular. The wound was deliberately left partially open (about 10 mm of visible wound) to allow for drainage, and a gauze dressing was applied to the small open area.

### Data collection

Age, sex, body mass index (BMI), comorbidities, American Society of Anesthesiologists (ASA) score, and risk factors for poor wound healing (e.g., diabetes mellitus, smoking) were noted. The underlying disease (benign vs. malignant), the interval from the primary surgery to stoma closure, and the type of stoma (ileostomy vs. colostomy) was noted. The primary endpoint was the rate of surgical site infection (SSI) in the short-term and the occurrence of postoperative hernia in the long-term period. Secondary endpoints included operative time, duration of wound dressing, incidence of reoperation, and surgical complications according to the Clavien–Dindo classification. The planned follow-up period for long-term complications was 5 years. In this study, Surgical Site Infection (SSI) was defined and classified strictly according to the Centers for Disease Control and Prevention (CDC) Guideline for the Prevention of Surgical Site Infection [14].

### Statistical analysis

All patient data were entered into an offline Microsoft Excel database. The normality of continuous variables was assessed using the Shapiro-Wilk test. Parametric variables are expressed as means ± standard deviation and were compared using Student’s t-test or ANOVA. Non-parametric variables are shown as medians (ranges) and were analyzed using the Mann-Whitney U test or Kruskal-Wallis test. Categorical data are presented as frequencies (%) and analyzed using Fisher’s exact test. A *p*-value < 0.05 was considered statistically significant. All analyses were performed using IBM^®^ SPSS^®^ Statistics for Macintosh, Version 28.0 (IBM Corp., Armonk, NY, USA).

## Results

There were no significant differences in baseline parameters between the VSC and PSC groups (Table 1). The median age was 66 years for both groups (PSC: 20–78 years; VSC: 42–76 years; *p* = 0.660). Male-to-female ratios, BMI, ASA scores, and underlying malignant vs. benign pathologies were comparable in both groups (all *p* > 0.05). The time from primary surgery to stoma closure was also similar (VSC: 210 [37–422] days vs. PSC: 187 [45–945] days, *p* = 0.791). Ileostomy closure was more frequent overall than colostomy closure (PSC: 55.3%, VSC: 67.6%, *p* = 0.345).

**Table 1:** Baseline characteristics of the study population

Parameter	Primary Closure (*n* = 38)	Volcano Technique (*n* = 37)	*p*-value
Type of stoma			0.345
Ileostoma	21 (55.3%)	25 (67.6%)	
Colostoma	17 (44.7%)	12 (32.4%)	
Age (years)	66 (20–78)	66 (42–76)	0.660
Sex			0.476
Male	26 (68.4%)	22 (59.5%)	
Female	12 (31.6%)	15 (40.5%)	
BMI (kg/m²)	26 (19.5–34.5)	28 (22–36)	0.067
Diabetes	7 (18.4%)	8 (21.6%)	0.779
Smoking	11 (28.9%)	11 (29.7%)	1.000
Dignity (Pathology)			0.626
Malignant	24 (63.2%)	26 (70.3%)	
Benign	14 (36.8%)	11 (29.7%)	
ASA score			0.712
ASA I	3 (7.9%)	2 (5.4%)	
ASA II	18 (47.4%)	21 (56.8%)	
ASA III	17 (44.7%)	14 (37.8%)	
Time to closure (days)	187 (45–945)	210 (37–422)	0.791
Operative time (min.)	87 (18–285)	59 (30–185)	0.034
Surgical Site Infection	18.4%	0.0%	0.012
Duration of dressing (days)	11 (8–23)	11 (6–23)	0.720
Hospital stay (days)	8 (3–18)	8 (5–15)	0.680

Source: Authors’ own construction

Two significant differences were observed: the median operative time was 59 min in the VSC group vs. 87 min in the PSC group (*p* = 0.034). Postoperative surgical site infection (SSI) occurred in 18.4% (7/38) of the PSC group but in 0% of the VSC group (*p* = 0.012).

**Fig. 1:**
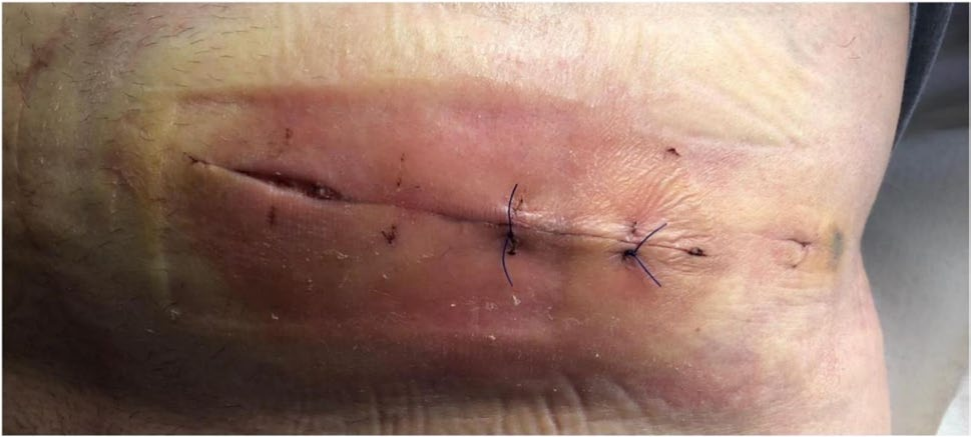
Wound healing disturbance four days after primary stoma closure

Of the seven PSC patients with SSI, five had Clavien-Dindo (CD) I complications requiring outpatient management for seroma or wound dehiscence (Fig. 1), while two required reoperation (CD III).

**Fig. 2:**
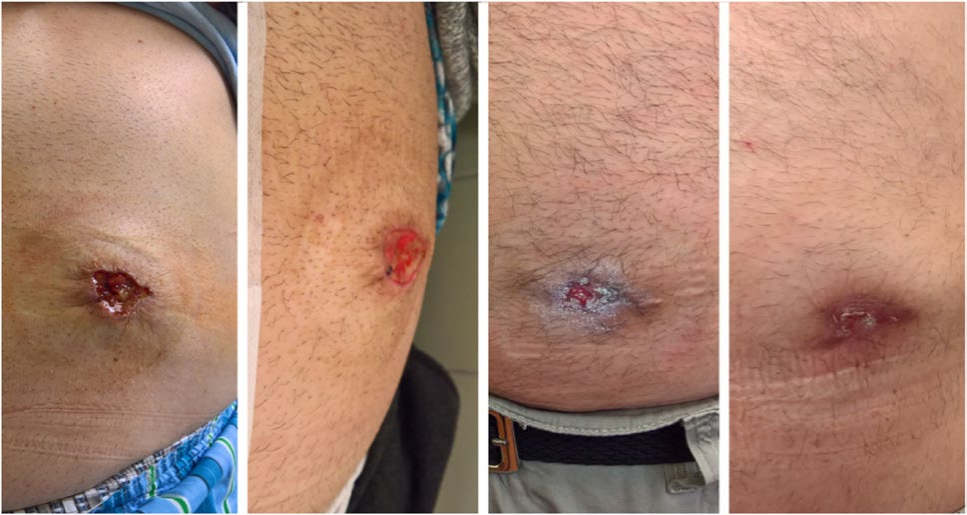
The healing process of a partially closed wound following the “Volcano” closure. The image on the right shows final healing 23 days after stoma reversal

No wound infections were reported among the VSC patients, although one patient in the VSC group did require surgery for adhesion-related ileus (CD III). There were no significant differences in length of hospital stay (PSC: 8 [3–18] days vs. VSC: 8 [5–15] days, *p* = 0.680) or duration of postoperative wound dressing (PSC: 11 [8–23] vs. VSC: 11 [6–23] days, *p* = 0.720) (Fig. 2).

### Follow-up

We planned a 5-year follow-up for the 75 patients enrolled in our study. Ultimately, 42 patients completed this follow-up, with pandemic-related challenges contributing to the reduced numbers. Of the 42 patients, a total of 13 developed a hernia at the stoma site (4 colostomy, 9 ileostomy), with hernia repairs being required in five cases. Among the patients who completed the follow-up, the incidence of parastomal hernias was notably lower in the Volcano Suture Closure group, with only 3 cases (7.14%) observed, compared to 10 cases (23.81%) in the Primary Suture Closure group. Six additional patients developed incisional hernias along the midline laparotomy (five post-colostomy, one post-ileostomy), with 3 cases observed in each group (VSC and PSC). Five patients were lost to follow-up due to the progression of their primary malignant disease.

## Discussion

Postoperative wound infection is an ongoing challenge in stoma takedowns, with various studies reporting SSI rates of up to 40% in some series [15–17]. Consistent with the literature, our study found an 18.4% SSI rate among patients undergoing primary closure, whereas no wound infections occurred in the Volcano (purse-string) group (*p* = 0.012). This aligns with prior studies by Marquez et al., Dusch et al., and Milanchi et al. [15–17], as well as a recent prospective randomized controlled study by Carannante et al. [18] all of which observed significantly lower or zero SSI rates with purse-string closure compared to primary closure. The total absence of infection in the VSC group fits the technique’s mechanism. Beyond simply minimizing subcutaneous dead space via circumferential approximation, the technique differs most significantly from primary closure by allowing continuous drainage through the central opening (approx. 5 mm). This small dehiscence prevents serosanguinous fluid accumulation, thereby avoiding the abscess formation associated with fully closed wounds. Our findings are further corroborated by a recent systematic review and meta-analysis by [19], which reported a 4% SSI rate in circular closure groups versus 27% in primary closure groups, reinforcing the strong preference for circular techniques to minimize infections. Additionally, [20] recently demonstrated that purse-string sutures are a vital component of surgical site infection reduction bundles, remaining effective even when combined with strict infection prevention protocols.

In addition to the low infection rate, the VSC group in our series had a significantly shorter operative time (59 vs. 87 min, *p* = 0.034). While some researchers found no difference or even longer operative times with purse-string closures [15, 17, 21], including Carannante et al. [18] who reported comparable operative times in their randomized trial, others have reported benefits similar to ours [16]. Surgeon familiarity with the technique and the extent of dissection required can strongly influence operating time. Although [13] noted that alternative techniques like the “gunsight” closure might offer higher patient satisfaction, they found no significant difference in SSI rates compared to purse-string methods, suggesting that the infection-control benefits of the technique used in our study remain robust.

Regarding hospital stay, we found no significant difference between the groups despite the reduction in SSI rates. While our study found no significant difference in the length of hospital stay or wound healing duration between the groups, recent literature suggests that adjunctive therapies may improve these specific metrics. A study by [22] demonstrated that combining purse-string closure with Negative Pressure Wound Therapy (NPWT) not only reduced SSI rates but also decreased postoperative pain and hospital stay. Similarly, [23] found that delayed primary closure combined with NPWT significantly reduced wound-healing duration compared to the purse-string method alone [24] also noted that NPWT reduced dressing change frequency. However, it is important to clarify that the length of stay in stoma reversal is primarily dictated by the recovery of bowel function (peristalsis and tolerance of oral intake) following the bowel anastomosis, rather than by the skin closure method alone. While the Volcano technique prevented wound infections, it did not accelerate the internal healing of the anastomosis, which remains the main determinant for discharge eligibility. Consequently, our results indicate that even without the added cost and complexity of NPWT, the standalone Volcano skin closure technique achieves superior infection control and operative efficiency, though the integration of NPWT could be considered in future cases to potentially accelerate the healing timeline.

We acknowledge certain limitations in our study. First, the study design was not randomized. Second, we lost approximately 44% of patients to 5-year follow-up, largely due to the disruptions caused by the COVID-19 pandemic. A further limitation is that this was a single-center study, which may affect the generalizability of our findings to other institutions with different surgical protocols.

## Conclusion

Our prospective analysis demonstrates that the purse-string “Volcano” closure significantly reduces the risk of surgical site infections (SSI) and, in our experience, shortens operative times compared to primary closure. These findings reinforce a growing consensus in recent literature that circular closure techniques offer superior perioperative outcomes. Consequently, we recommend the Volcano technique as a standard approach for both ileostomy and colostomy reversals.

Future research should prioritize randomized trials comparing VSC with alternative methods like the gunsight technique, specifically focusing on cosmesis, patient satisfaction, and quality of life. Furthermore, investigating the integration of negative-pressure wound therapy (NPWT) could provide valuable insights into accelerating wound healing and shortening hospital stays in high-risk patients.

## Supplementary Information

Supplementary Material 1.

## Data Availability

Yes, I have research data to declare. “Included in the paper or Supplementary Information”.
